# Multiantenna NOMA with Finite Blocklength: A Pragmatic Paradigm for Ultra-Dense Networking

**DOI:** 10.3390/e28030281

**Published:** 2026-03-01

**Authors:** Haoming Wang, Zhenzhen Zhang, Xinhao Wu, Bing Li

**Affiliations:** 1 School of Software, Northwestern Polytechnical University, Xi’an 710129, China; 2 Powerchina Central China Electric Power Engineering Co., Ltd., Zhengzhou 450007, China; 3 GuangZhou Great Power Energy & Technology Co., Ltd., Guangzhou 511483, China

**Keywords:** low power wide area network, massive connectivity, multi antenna, nonorthogonal multiple access, ultra reliability

## Abstract

This paper addresses the design and performance analysis of nonorthogonal multiple access (NOMA) for ultra-dense networking of the Internet of Things (IoT) based on low-power sensors. The proposed NOMA schemes consist of an $N_{r}$-antenna access point and *K* single antenna sensors given $K\gg N_{r}$. A power allocation technique and forward error correction (FEC) are combined to enable concurrent uplink transmission and the successful separation of all *K* sensors at the access point. In scenarios where $K\gg N_{r}$, large dimensional analysis is employed to derive a deterministic expression for the received signal-to-interference-plus-noise ratio (SINR) within the finite blocklength regime. Three distinct Forward Error Correction (FEC) codes—convolutional codes (CCs), polar codes, and low-density parity-check codes (LDPCs)—are assessed. These evaluations indicate that all three codes achieve near-capacity performance while supporting massive connectivity in the finite-blocklength context. Notably, convolutional codes demonstrate comparable performance with reduced complexity, a desirable attribute for prolonging the life cycle of wireless sensor network-based IoT applications.

## 1. Introduction

The Internet of Things (IoT) is regarded as a disruptive enabler in 5G and beyond, facilitating massive machine-type communications (mMTC) and enabling seamless networking of diverse entities [1]. A prominent challenge in mMTC is efficiently utilizing limited resources while supporting ultra-dense networking and achieving massive connectivity. To address this, nonorthogonal multiple access (NOMA) has been developed [2] and successfully applied in a number of scenarios, including ultra-reliable low-latency communication (URLLC) healthcare systems [3], smart industry [4], and vehicular networks [5].

As a particular instance of superposition transmission, NOMA effectively accommodates multiple users within a single time-frequency resource block (i.e., channel) to considerably enhance data rates and URLLC performance. Nevertheless, it is apparent that this approach requires sophisticated design to mitigate the substantial multiuser interference and ensure successful user separation. Power domain NOMA is one of several design strategies that have been thoroughly examined, cf. [6]. The essence of power-domain NOMA is that users are allocated distinct transmit power levels and are subsequently detected using successive interference cancellation (SIC) at the receiver. Both analytical and experimental results indicate that power-domain NOMA significantly improves spectral efficiency compared to orthogonal multiple-access (OMA) methods. Moreover, power-domain NOMA can provide physical-layer security in both uplink and downlink networks. In this application, friendly jammer or artificial interference techniques are utilized [7,8] such that wiretapping links can barely be established among targets and eavesdroppers.

Conversely, code domain NOMA primarily employs forward error corrections (FECs) to mitigate interference [9]. The FEC range spans from simple convolutional codes (CC) to advanced low-density parity-check codes (LDPC) and polar codes. By leveraging strong error correction capabilities, multiuser interference is reduced. Despite this, various NOMA variants effectively support extensive connectivity. However, challenges arise when a stringent quality of service (QoS) in terms of reliability, latency, and massive connectivity is required. Recently, significant attention has been directed toward addressing these challenges, with NOMA based on finite blocklength transmission (FBT) emerging as a viable technique to enable URLLC.

### 1.1. Previous Works

The FBT theory was initially comprehensively examined in [10], and subsequently integrated into NOMA to enhance performance in URLLC scenarios, as illustrated in [11,12,13,14]. An early work focusing on NOMA-based FBT, i.e., [11], addresses the downlink URLLC transmission of NOMA. The analysis and results show that NOMA outperforms OMA in terms of spectral efficiency and latency in the finite blocklength regime. In [12], the delay performance of single-antenna uplink transmission under conditions of imperfect channel state information is examined. A closed-form expression for the achievable spectral efficiency is derived. In [13], a protocol stack is proposed and evaluated to enhance the performance of body area networks, assuming concurrent transmission by two nodes employing binary phase-shift keying (BPSK) modulation. Enhanced spectral efficiency and reduced latency of NOMA are verified. In [14], the performance of uplink finite blocklength transmission–nonorthogonal multiple access (FBT-NOMA) is assessed in terms of hardware impairments and channel estimation errors. Analytical expressions are derived and subsequently validated through experimental results. Further enhancements are achieved by integrating multiple antenna techniques with NOMA, as demonstrated in [15,16], where multi-antenna FBT-NOMA (mFBT-NOMA) is developed and thoroughly examined.

The essence of the aforementioned scheme is using a multi-antenna beamforming technique to generate mutually orthogonal beam-spaces, each capable of accommodating multiple users when power allocation is applied. Consequently, NOMA supports a significantly greater number of users. There is no doubt that power allocation plays a key role in NOMA. In [15], the techniques of downlink beamforming and signal alignment are employed and subsequently analyzed using stochastic network calculus. The approach in [16] examines mFBT-NOMA utilizing the channel inversion power allocation algorithm, highlighting the trade-off between reliability and latency. In [17,18,19], combinatorial optimization is proposed to generate power allocation in massive connectivity scenarios, which offers improved efficiency.

### 1.2. Motivations and Contributions

Although the NOMA code domain has attracted extensive attention, the application of FBT still needs further development. The code domain NOMA treats interference as additive white Gaussian noise (AWGN) in principle, which can be mitigated using powerful FECs such as CC, LDPC, and polar codes. Such a method perhaps dates back to [20,21], and some recent results can be found in, e.g., [22,23]. While supporting massive connectivity, as power-domain NOMA does, the blocklength required approaches infinity to achieve near-capacity performance and successful interference removal. The performance may degrade significantly as the blocklength is reduced to a few thousand bits in both single-antenna [23] and multi-antenna systems. It is thus desirable to optimize the performance of FBT-NOMA in the code domain to enable URLLC.

Another challenge that arises in next-generation NOMA is the construction of NOMA when practical waveforms are considered [24]. Most existing NOMA schemes assume ideal Gaussian waveforms to facilitate design and performance analysis, whereas the interference is also Gaussian-distributed as a result. However, the implementation of NOMA is primarily based on practical waveforms, including BPSK [24]. This challenge, unfortunately, bottlenecks the implementation of NOMA based on pivotal IoT standards, namely IEEE 802.15.4, Bluetooth, and IEEE 802.15.6 [25], where BPSK, minimum shift keying (MSK), and Gaussian MSK (GMSK) are employed. As discussed in some recent works [26,27,28,29,30], practical waveforms based NOMA schemes are appealing but may hardly offer the performance when an ideal Gaussian waveform is used. An acceptable explanation is that using FEC or power allocation alone is incapable of mitigating non-Gaussian interference [22]. This issue becomes more problematic when nonlinear waveforms, i.e., MSK/GMSK, are used.

This paper attempts to design MSK/GMSK-based mFBT-NOMA to enable massive connectivity IoT network. The NOMA method is adopted such that an access point (AP) equipped with $N_{r}$ antennas serves *K* single antenna users given $N_{r}\ll K$, to avoid severe absorption due to high frequency [31] or large size antenna array due to industrial, scientific, and medical (ISM) band. To achieve these objectives, the text addresses two significant topics. Firstly, the performance concerning the achievable rate is examined by assessing the attainable signal-to-interference-plus-noise ratio (SINR) within a massive connectivity framework. Design guidelines are subsequently derived from this analysis. Secondly, a practical mFTB-NOMA system based on MSK/GMSK is developed, and the guidelines are thoroughly validated, resulting in the identification of a low-complexity transceiver suitable for IoT.

While this paper focuses on the design and optimization of NOMA for massive IoT connectivity in the finite blocklength regime, the principles and techniques developed herein may find applications in emerging applications such as integrated sensing and communications (ISAC) [32] and physical layer security [7,8], where NOMA has demonstrated promising potential. Such cross-fertilization of ideas between IoT connectivity and ISAC represents an exciting direction for future research.

The contributions of this paper are mainly as follows:

1. Combination of FEC and power allocation for NOMA. As implied in [16], the power allocation of mFBT-NOMA is challenging since a closed-form expression of SINR is not easy to derive. This paper utilized high-dimensional matrix analysis to demonstrate that the SINR of mFBT-NOMA converges to a deterministic and interpretable value, such that a recursive power allocation (RPA) scheme is made possible, taking SIC into consideration. This technique avoids exquisite but complex expression required in most existing schemes, see, e.g., [14,15,16].
2. Overloaded NOMA in FBL regime. In most power-domain NOMA systems, a multi-antenna scheme enables $K=2N_{r}$ users to share the channel, where $N_{r}$ clusters are formed, and each cluster accommodates two users, commonly referred to as near and far users. Such a design significantly alleviates inter-cluster interference, and power allocation is largely concentrated on the two users within each cluster [6]. In contrast, the proposed mFBT-NOMA can support up to K=40 users with only $N_{r}=6$, thereby achieving a substantially higher overload factor than conventional power-domain NOMA schemes, without resorting to clustering techniques. To mitigate the resulting severe interference, 5G forward error correction (FEC) codes are employed. Analytical and numerical results demonstrate that both ultra-reliable low-latency communications (URLLC) and massive connectivity are enabled, thereby validating the effectiveness of the mFBT-NOMA methodology.
3. Low complexity transceiver for nonlinear waveforms. In addition to the analysis mentioned above, the proposed design is exemplified using mFBT-NOMA, which consists of MSK/GMSK waveform and 5G URLLC FECs candidates, including CC, LDPC, and polar code. Simulated results confirm that the proposed schemes achieve the targeted reliability and massive reliability in the finite block length regime. Moreover, the proposed scheme is demonstrated to outperform LDPC- and BPSK-based mFBT-NOMA [33], which is a well-established standard.

The rest of this paper is organized as follows. Section 2 presents the system model; Section 3 presents the method proposed for design and performance analysis; Section 4 presents the numerical results concerning different FECs in terms of connectivity and spectral efficiency given various configurations. Section 5 concludes the paper.

## 2. System Model and Problem Formulation

**Figure 1:**
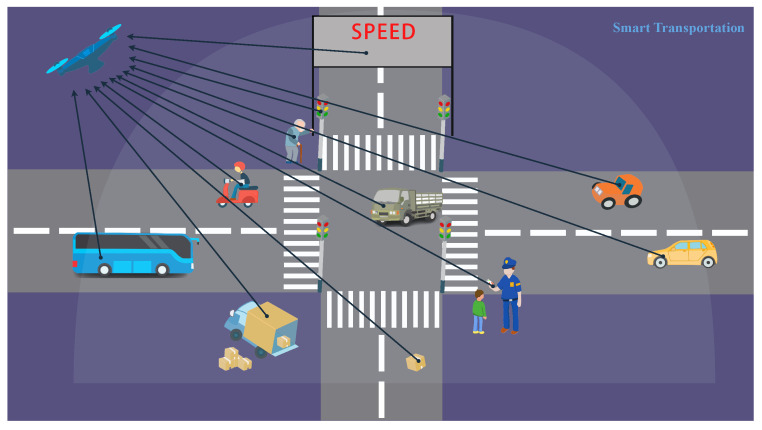
Drone-aided IoT networking scenario.

As presented in Figure 1, this paper concerns the uplink transmission of an IoT network using mFBT-NOMA, where an $N_{r}$-antenna AP serves *K* single-antenna sensors. The transmitter consists of three components, namely FEC, user-specific interleaver $\Pi_{k}$, and MSK/GMSK modulator (Mod), while the receiver consists of three components, namely linear minimum mean square error (LMMSE) filter for interference suppression, MSK/GMSK demodulator (DMod), and FEC decoder (DEC) accordingly.

### 2.1. System Model and Transceiver

**Figure 2:**
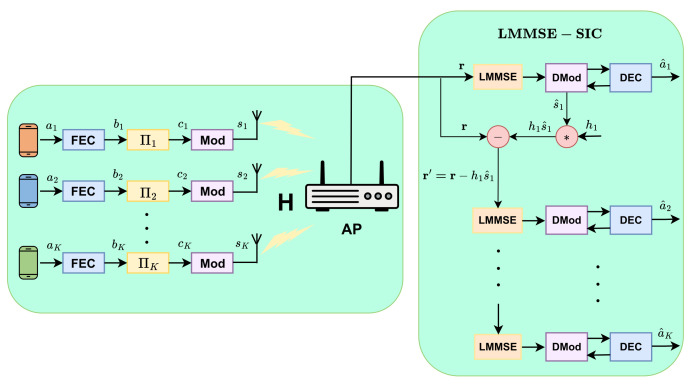
mFBT-NOMA system model.

The system model is then quantitatively depicted in Figure 2. Taking user-*k* as an example, the workflow of the transceiver is outlined briefly as follows. The *L*-bits-long information bit $a_{k}$ is firstly encoded to generate *N*-bits long coded $b_{k}$ and then permutated to form bits $c_{k}$ input to the MSK/GMSK modulator. The resultant signal reads

(1) $$s\left(t,c_{k}\right)=\sqrt{p_{k}}\exp\left[\phi \left(t,c_{k}\right)+\phi_{k}\right]$$

where $p_{k}$ is the transmit power of user-*k*, $\phi (t,c_{k})$ is the information-bearing phase, and the initial phase $\phi_{k}$ is defined as

(2) $$\phi_{k}=k\times \Delta \phi$$

where Δϕ is calculated in advance to facilitate the design and user separation [34]. The information-bearing phase $\phi (t,c_{k})$ is detailed as

(3) $$\phi \left(t,c_{k}\right)=\pi \sum_{i=1}^{n-L}c_{k}\left(i\right)+2\pi h\sum_{i=n-L+1}^{n}c_{k}\left(i\right)q\left(t-iT\right)$$

where n∈[1,N] is the time index, *T* is the symbol duration; *h* is the modulation index, which equals 0.5 throughout this paper; and *L* indicates the memory of modulation. The function q(t) is the phase response, and its frequency pulse g(τ) is the derivative of q(t). It is assumed that g(τ) is of finite duration, occupying the interval [0,LT]. If g(τ) has support [0,T] or less, the scheme is a full response (L=1); otherwise, it is a partial response scheme. For normalization purposes, g(τ) integrates to 1/2 whenever it has a nonzero integral. Equivalently, q(t) satisfies q(t)=0,t<0. In the case of the MSK waveform. g(τ) reads

(4) $$g\left(\tau \right)=\frac{1}{2LT}$$

Similarly, for GMSK, a different expression is adopted, i.e.,

(5) $$g\left(\tau \right)=\frac{1}{2T}\left[Q\left(\frac{2\pi B}{\sqrt{\ln2}}\left(\tau -\frac{T}{2}\right)\right)-Q\left(\frac{2\pi B}{\sqrt{\ln2}}\left(\tau +\frac{T}{2}\right)\right)\right]$$

where *B* is a bandwidth configure parameter and Q(x) is the so-called Gaussian *Q*-function defined as

(6) $$Q\left(x\right)=\int_{x}^{\infty }\frac{1}{\sqrt{2\pi }}\exp\left(-\frac{y^{2}}{2}\right)dy.$$

Due to the continuous and smooth phase variation (3), MSK and GMSK offer compact power spectral density (PSD). As a result, the normalized bandwidths of MSK and GMSK are 1.0 and 1.2, respectively. To enable low complexity detection, principal component analysis (PCA) [35] is employed at the receiver side. Hence $s(t,c_{k})$ admits an equivalent and discrete representation $s\left(t,c_{k}\right)\overset{\mathrm{PCA}}{\to }s_{k}$, and the received signal r is written as

(7) r=Hs+n

where $s={\left[s_{1},\cdots ,s_{K}\right]}^{T}$ collects signals from all users; $H={\left[h_{ij}\right]}_{N_{r}\times K}$ represents the channel state information (CSI) matrix and the elements $h_{ij}$ are independent and identically distributed (i.i.d) complex Gaussian random variables, i.e., ∀i,j, $h_{ij}∼\mathrm{CN}\left(0,I\right)$; $n={\left[n_{1},\cdots ,n_{N_{r}}\right]}^{T}$ stands for AWGN and $\forall m=1,\cdots ,N_{r}$, $n_{m}∼\mathrm{CN}\left(0,1\right)$.

Obviously, the received signal is interference-limited as K≫1. To suppress the severe multiuser interference, user *k* firstly processes r by an LMMSE filter $l_{k}$ expressed as

(8) $$l_{k}=\sqrt{p_{k}}h_{k}^{†}\left(\sum_{m=1}^{K}p_{m}h_{m}h_{m}^{†}+I\right)$$

where $h_{k}$ is the *k*-th column of H, standing for the CSI vector between AP and user *k*; † stands for Hermitian conjugate operation.

As a result, the signal power after the LMMSE filter is

(9) $${\left[p_{k}h_{k}^{†}{\left(\sum_{m=1}^{K}p_{m}h_{m}h_{m}^{†}+I\right)}^{-1}h_{k}\right]}^{2}$$

and, after some manipulation, the interference power can be expressed as

(10) $$p_{k}h_{k}^{†}{\left(\sum_{m=1}^{K}p_{m}h_{m}h_{m}^{†}+I\right)}^{-1}h_{k}-{\left[p_{k}h_{k}^{†}{\left(\sum_{m=1}^{K}p_{m}h_{m}h_{m}^{†}+I\right)}^{-1}h_{k}\right]}^{2}$$

Hence the SINR $\gamma_{k}$ can be expressed as

(11) $$\begin{array}{cc} \gamma_{k} & =\frac{p_{k}h_{k}^{†}{\left(\sum_{m=1}^{K}p_{m}h_{m}h_{m}^{†}+I\right)}^{-1}h_{k}}{1-p_{k}h_{k}^{†}{\left(\sum_{m=1}^{K}p_{m}h_{m}h_{m}^{†}+I\right)}^{-1}h_{k}}\\ \; & =p_{k}h_{k}^{†}{\left(\sum_{m\neq k}p_{m}h_{m}h_{m}^{†}+I\right)}^{-1}h_{k} \end{array}$$

where the last line is obtained using matrix inversion lemma $x^{†}{\left(A+\alpha xx^{†}\right)}^{-1}x=\frac{x^{†}A^{-1}x}{1+\alpha x^{†}A^{-1}x}$. Upon reception of the filtered signal, the MSK/GMSK demodulator obeys the maximum a posteriori probability (MAP) principle [36] to output extrinsic information to the FEC decoder, which is also a MAP module. The exchange of extrinsic information between demodulator and decoder carries out $i_{\max}$ times and generates ${\hat{a}}_{k}$ and ${\hat{s}}_{k}$, which are the estimation of information bits and transmitted signal of user *k*, respectively. Then ${\hat{s}}_{k}$ is subtracted from r, and the resultant quantity $r^{\prime }=r-h_{k}{\hat{s}}_{k}$ is sent to user k+1 for LMMSE filtering, demodulator, and decoder.

The above procedure is repeated for all users indexed by k∈[1,K] and is termed LMMSE successive interference cancellation (LMMSE-SIC).

### 2.2. Problem Formulation

As mentioned earlier, this paper focuses on support of massive uplink connectivity in finite blocklength regime [10]. Given finite blocklength *N*, the achievable spectral efficiency $R_{k}$ (bits/s/Hz) is calculated as

(12) $$R_{k}\approx R\left(N,R_{k},\epsilon \right)=\frac{1}{2}\log_{2}\left(1+\gamma_{k}\right)-\sqrt{\frac{V}{N}}Q^{-1}\left(\epsilon \right)$$

where $\gamma_{k}$ is the SINR of user *k* (11), ϵ is the target block error rate (BLER), $V=\frac{\log_{2}^{2}e}{2}\left[1-\frac{1}{{\left(1+\gamma_{k}\right)}^{2}}\right]$, and Q(x) is the Gaussian *Q*-function defined in (6). The expression (12) is extensively leveraged to facilitate the design and performance analysis of mFBT-NOMA in, e.g., [12,13,14,15,16].

Apparently, (12) is SINR-centric. However, $\gamma_{k}$ is essentially a random variable due to $h_{k},k\in \left[1,K\right]$ as implied by (11). Existing methods mainly focus on two-user setups to obtain a tractable expression, though the result is frequently based on approximation, see e.g., [14,15]. Hence, this method may turn out to be infeasible in the context of massive connectivity, where the number of concurrent users K≫2 as concerned in this paper.

## 3. Design and Performance Analysis

Before presenting the details, some assumptions must be made. First, it is assumed that all users attain the same spectral efficiency $R=R_{k}$, ∀k∈[1,K] to enable fairness among all users. To enable *R*, the minimum feasible value of $\gamma_{k}$ is

(13) $$\gamma_{\mathrm{th}}=R^{-1}\left(N,R,\epsilon \right)$$

Secondly, it is assumed that $p_{1}>p_{2},\cdots ,p_{k}$ such that LMMSE-SIC can successfully detect all users as adopted in NOMA, and hence user *k* is subject to interference from users indexed by m∈[k+1,K], which is well justified in theory [6] and practice [14,15]. A direct result of this assumption is that $\gamma_{k}=p_{k}h_{k}^{†}{\left(\sum_{m>k}p_{m}h_{m}h_{m}^{†}+I\right)}^{-1}h_{k}$, where the interference has been removed using LMMSE-SIC.

### 3.1. Asymptotic Convergence of $\gamma_{k}$

As pointed out earlier, $\gamma_{k}$ is a random variable. Fortunately, some recent results indicate that $\gamma_{k}$ converges to its expectation $\mu_{k}=E\left(\gamma_{k}\right)$ almost surely. This result is utilized to obtain a closed-form expression for $\gamma_{k}$.

Starting with (12), $\gamma_{k}$ can be rewritten as [3,37]

(14) $$\begin{array}{cc} \gamma_{k} & =p_{k}h_{k}^{†}{\left(\sum_{m>k}p_{m}h_{m}h_{m}^{†}+I\right)}^{-1}h_{k}\\ \; & =\frac{p_{k}}{p_{a}}\;\cdot \;p_{a}h_{k}^{†}{\left(\sum_{m>k}p_{a}h_{m}h_{m}^{†}+I\right)}^{-1}h_{k} \end{array}$$

where the (14) is from ([38], (46)) given $\sum_{m<k}p_{m}=\left(k-1\right)p_{a}$. Intuitively, this result implies that ${\left(\sum_{m>k}p_{a}h_{m}h_{m}^{†}+I\right)}^{-1}$ is numerically equivalent to ${\left(\sum_{m>k}p_{m}h_{m}h_{m}^{†}+I\right)}^{-1}$, since the total interference strength matters rather than the specific value of $p_{m}$, which has been the *de facto* method in multiuser theory. Unfortunately (14) is still intractable, further simplification can be made as $K,N_{r}\to \infty$

(15) $$\gamma_{k}\overset{a.s}{\to }\mathrm{Mean}\left(\gamma_{k}\right)=\frac{p_{k}}{p_{a}}\;\cdot \;N_{r}p_{a}\left(1-\frac{F\left(N_{r}p_{a},\beta \right)}{4N_{r}p_{a}}\right)$$

where a.s stands for almost sure convergence, $\beta =\left(K-k+1\right)/N_{r}$, and the function $F\left(x,y\right)$ admits a closed-form expression

(16) $$F\left(x,y\right)={\left(\sqrt{x{\left(1+\sqrt{y}\right)}^{2}+1}-\sqrt{x{\left(1-\sqrt{y}\right)}^{2}+1}\right)}^{2}$$

which is the η-transform of the Mar$\overset{˘}{c}$enko–Pastur distribution ([39], Theorem 2.39) and hence

(17) $$\gamma_{k}\overset{a.s}{\to }\alpha_{k}\mu_{k}$$

where $\alpha_{k}=p_{k}/p_{a}$ and $\mu_{k}=\frac{1}{\beta -1}$ ([39], Section 3.1.1). Due to this convergence, it is reasonable to replace $\gamma_{k}$ with $\alpha_{k}/\left(\beta -1\right)$ in (12) to obtain a simple yet tractable expression.

Moreover, such a replacement is rather accurate, seeing that the variance

(18) $$\mathrm{Var}\left(\gamma_{k}\right)=E{\left(\gamma_{k}-\frac{\alpha_{k}}{\beta -1}\right)}^{2}=\alpha_{k}^{2}\;\cdot \;V\left(\mu_{k},\beta ,p_{a}\right)$$

where $V(\mu_{k},\beta ,p_{a})$ is expressed as ([40], Theorem 4.5)

(19) $$V\left(\mu_{k},\beta ,p_{a}\right)=\frac{1}{N_{r}}\left[\frac{2{\left(1+\mu_{k}\right)}^{2}\mu_{k}p_{a}}{{\left(1+\mu_{k}\right)}^{2}+\beta p_{a}}-\mu_{k}^{2}\right]$$

as $N_{r}\to \infty$ and limited $p_{a}$ and hence $\mu_{k}$, $V(\mu_{k},\beta ,p_{a})\to 0$ as a result. It turns out that the replacement is reliable and accurate.

Unfortunately, the results regarding $\gamma_{k}$ show little line on power allocation, since it is determined by $p_{a}$ instead of $p_{k}$. To this end, the following lemma is presented.

**Lemma** **1.** *LMMSE filter improves the SINR by a factor of $N_{r}^{2}/{∥h_{k}∥}_{F}^{2}$ approximately, given $K,N_{r}\to \infty$ and β≫1.*

**Proof.**  The improvement can be expressed as

(20) $$\frac{\alpha_{k}\mu_{k}}{\frac{p_{k}{∥h_{k}∥}_{F}^{2}}{\sum_{m>k}p_{m}{∥h_{m}∥}_{F}^{2}+1}}\approx \frac{N_{r}^{2}}{∥h_{k}{∥}_{F}^{2}}=N_{\Delta }$$

where $p_{k}{∥h_{k}∥}_{F}^{2}/\left(\sum_{m>k}p_{m}∥h_{m}{∥}_{F}^{2}+1\right)$ is the SINR of the received signal before applying LMMSE. Neglecting the contribution from AWGN and seeing $∥h_{m}{∥}_{F}^{2}\to N_{r}$, the right-hand side (RHS) is obtained as long as $K-k\gg N_{r}$ just as assumed. The factor $N_{\Delta }$ is interpreted as the diversity gain when only knowing $h_{k}$ but not $h_{m}$. As a result, the transmit power $p_{k}$ can be reduced by a factor of $N_{\Delta }$ to obtain the expected $\gamma_{k}$. □

### 3.2. RPA for mFBT-NOMA

Given Lemma 1, RPA is presented below. Assuming the target SINR is $\gamma_{\mathrm{th}}$ for all users, as a consequence of the fairness assumption. The algorithm starts from user *K*, the weakest user. That is, for user *K*, the power allocated is $p_{K}=\gamma_{\mathrm{th}}/N_{\Delta }$ since the AWGN is of unit strength. For k<K, it can be shown that a recursive expression holds, i.e.,

(21) $$p_{k}=\gamma_{\Delta }{\left(1+\gamma_{\Delta }\right)}^{K-k},\forall 1\leq k\leq K$$

where $\gamma_{\Delta }=\gamma_{\mathrm{th}}/N_{\Delta }$, and hence the average power of the interfering users indexed by m<k is

(22) $$p_{a}=\frac{\gamma_{\Delta }\left[1-{\left(1+\gamma_{\Delta }\right)}^{K-k}\right]}{\left(K-k\right)\left[1-\left(1+\gamma_{\Delta }\right)\right]}\approx \frac{{\left(1+\gamma_{\Delta }\right)}^{K-k}}{K-k}$$

where the RHS is obtained since $\left(1+\gamma_{\mathrm{th}}/N_{\Delta }\right)>1$, and therefore according to (17) and (21)

(23) $$\gamma_{k}=\frac{p_{k}}{p_{a}}\;\cdot \;\frac{1}{\beta -1}=\gamma_{\mathrm{th}}\left(\frac{K-k}{K-k+1-N_{r}}\right)\geq \gamma_{\mathrm{th}}$$

as long as $K-k+1\geq N_{r}$. When $K-k+1<N_{r}$, a closed-form expression is hardly available for this underloaded system since large dimensional analysis would be out of function, and the method presented in [15,16] can be used.

### 3.3. Fairness and Power Consumption

To ensure fairness among users in terms of attainable spectral efficiency, it is assumed that all users achieve *R* simultaneously. This method is equivalent to the max-min fairness principle as presented in ([41], Theorem 1), which pointed out that the fairness is established as long as the following criterion holds

(24) $$\gamma_{k}\geq 2^{2R}-1⟷\alpha_{k}\mu_{k}\geq \gamma_{\mathrm{th}}$$

where the RHS is obtained from (17) and (12), which automatically hold due to the result presented in (23).

The power consumption may be a concern, given that some users are much stronger than the rest, as implied by (21). This issue is resolvable in the long term because a user can be assigned different power levels from time to time without affecting performance, as $\gamma_{k}$ is designed to do. As a matter of fact, this long-term fairness can be guaranteed by resorting to the random access protocol presented in [42]. As a result, users’ power consumption eventually tends to be identical, and the sustainability of IoT is not undermined.

### 3.4. Accuracy Analysis

**Figure 3:**
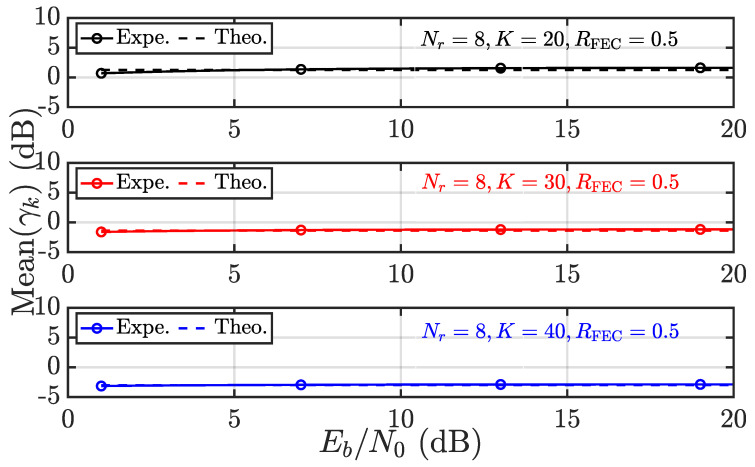
Given $\alpha_{k}=2$ and varying *K*, the $\mathrm{Mean}(\gamma_{k})$ is evaluated in terms of experimental (Expe.) results vs. theoretical (Theo.) results according to (17).

**Figure 4:**
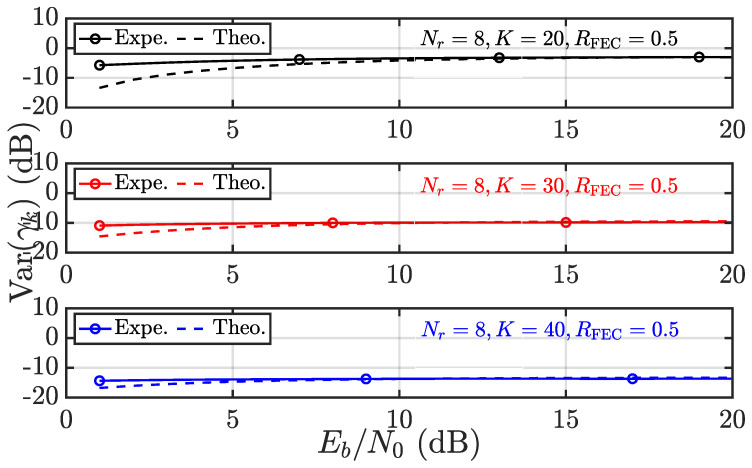
Given $\alpha_{k}=2$ and varying *K*, the $\mathrm{Var}(\gamma_{k})$ is evaluated in terms of experimental (Expe.) results vs. theoretical (Theo.) results according to (18).

**Figure 5:**
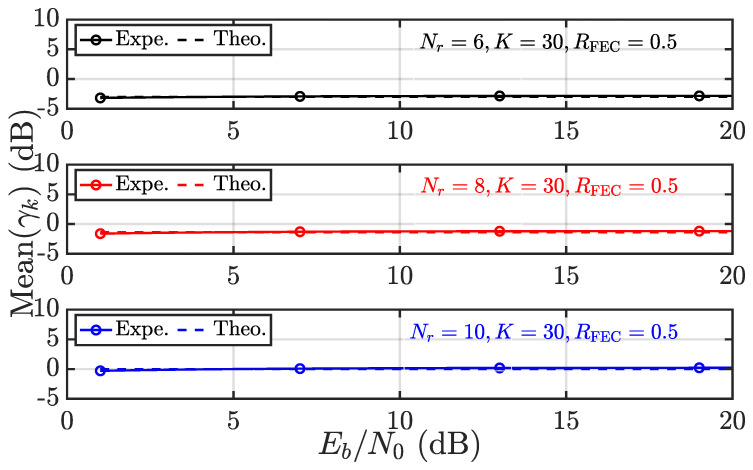
Given $\alpha_{k}=2$ and varying $N_{r}$, the $\mathrm{Mean}(\gamma_{k})$ is evaluated in terms of experimental (Expe.) results vs. theoretical (Theo.) results according to (17).

**Figure 6:**
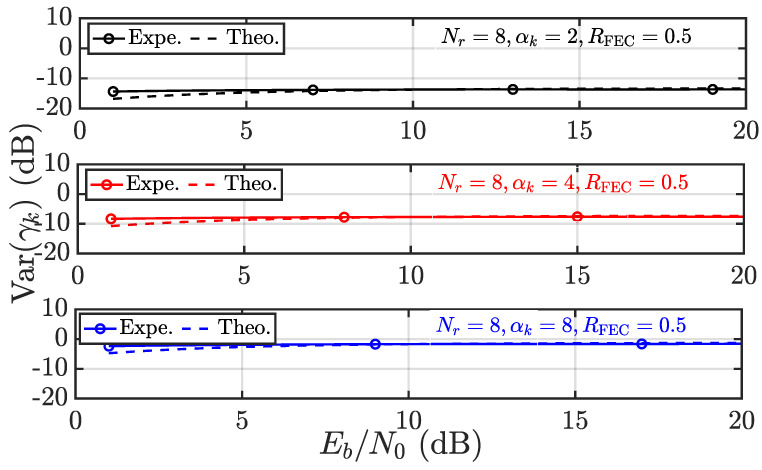
Given K=40 and varying $\alpha_{k}$, the $\mathrm{Var}(\gamma_{k})$ is evaluated in terms of experimental (Expe.) results vs. theoretical (Theo.) results according to (18).

The foregoing discussion is dedicated to the convergence behavior of $\gamma_{k}$, which serves as a guarantee of RPA and fairness. It is thus wondered about the accuracy regarding the results obtained, especially (17) and (18). To this end, the experimental results and theoretical results from (17) and (18) are compared in Figure 3, Figure 4, Figure 5 and Figure 6. Throughout this subsection, user 1 (i.e., k=1) is considered, and $p_{a}$ as the average power of interference is expressed in terms of $E_{b}/N_{0}$ to take into account the code rate of FECs denoted as $R_{\mathrm{FEC}}$.

In Figure 3 and Figure 4, the systems with $N_{r}=8$ and K=20,30,40 are considered. First, in Figure 3, the mean of $\gamma_{k}$ is evaluated theoretically and experimentally as $E_{b}/N_{0}$ varies from 0 dB to 20 dB. Overall, the experimental and theoretical results coincide very well, regardless of the user load *K*. The identical systems are then evaluated in terms of $\mathrm{Var}\left(\gamma_{k}\right)$ in Figure 4. It is observed that the experimental and theoretical results agree very well, except in the low $E_{b}/N_{0}$ region. That being said (19) appears to underestimate $\mathrm{Var}(\gamma_{k})$ in the low $E_{b}/N_{0}$ region. The difference vanishes with increasing $E_{b}/N_{0}$. Such a phenomenon is a result of making $N_{r}=8$ and K≤40, rather than $N_{r},K\to \infty$. Fortunately, $\mathrm{Var}(\gamma_{k})$ (in dB) is always negative and reaches nearly −20 dB as K=40, validating the almost sure convergence behavior of $\gamma_{k}$ with increasing size.

The $\mathrm{Mean}(\gamma_{k})$ and $\mathrm{Var}(\gamma_{k})$ are also evaluated given varying $N_{r}$ and $\alpha_{k}$, where the results confirm (17) and (18) again. Further, it is observed in Figure 5 that increasing $N_{r}$ leads to increasing $\gamma_{k}$ as a result of diversity gain. However, it is also noticed that increasing $\alpha_{k}$ would result in significantly increased $\mathrm{Var}(\gamma_{k})$, which is also indicated by the factor $\alpha_{k}^{2}$.

### 3.5. FECs in mFBT-NOMA

The discussion has been completely SINR-centric so far, which does not necessarily lead to perfect interference mitigation in practice from an information-theoretic perspective, posing a major challenge and even disproving the assumption presented at the beginning of this section. This challenge is frequently referred to as SIC error propagation [6].

This challenge motivates the employment of FEC to mitigate the residual interference due to imperfect SIC as extensively justified in [9] and references therein. In this paper, three different FECs are considered, namely CC, LDPC, and polar, which are all 5G candidates [43]. Among them, CC is widely adopted in IoT [1] due to the low implementation complexity, while the other two are renowned for capacity-approaching performance with prohibitively high complexity for IoT [1,43]. The CC recommended is ${\left(7,5\right)}_{8}$, a simple 4-state CC that has been extensively studied [30,36,42] when MSK and GMSK modulations are used and has been proven to offer near-capacity performance over single-antenna AWGN channels. The parameters of FECs are listed in Table 1 below.

Here, the complexity is summarized qualitatively, and a quantitative description in terms of chip area can perhaps be found in ([43], Figure 10). The block length *N* is limited to 512 or 1024 to enable URLLC as aforementioned. To keep decoding complexity as low as possible, all decoding algorithms are defined in the log domain. The algorithms are soft-input soft-output algorithms, i.e., MAP, belief propagation (BP), and soft cancellation (SCAN) for CC, LDPC, and polar codes, which facilitate fair comparison among FECs but may lead to increased BLER.

## 4. Numerical Results

In this section, the proposed design is validated using MSK-/GMSK-based coded modulations in terms of actual attainable BLER, spectral efficiency *R*, and connectivity *K* given the FECs presented in Table 1. When MSK (or GMSK) is used, the achievable spectral efficiency *R* is 0.417 bps (or 0.5 bps) due to a normalized bandwidth being 1.2 (or 1.0) as $R_{\mathrm{FEC}}=0.5$. The target BLER is $\epsilon =10^{-5}$ in the subsequent discussion.

**Figure 7:**
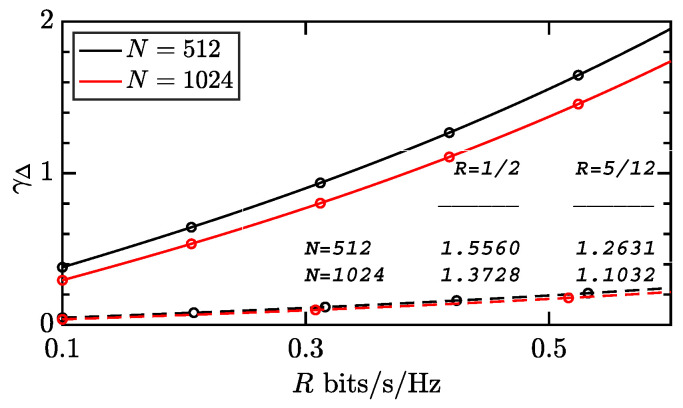
The required $\gamma_{\Delta }$ given N=512(blacklines),1024(redlines), R=1/2bps(solidlines), 5/12bps(dashedlines), and $\epsilon =10^{-5}$ in (13).

First, in Figure 7, the required $\gamma_{\Delta }$ in (21) is calculated. Two cases are considered for conciseness, i.e., $N_{r}=1$ and $N_{r}=8$. The quantity $\gamma_{\mathrm{th}}$ is also tabulated in the figure, such that $\gamma_{\Delta }=\gamma_{\mathrm{th}}/N_{r}$ is readily obtained for arbitrary $N_{r}$. In general, using multiple antennas can significantly improve energy efficiency due to the reduced $\gamma_{\Delta }$, as can be seen in (22). When given the same $N_{r}$, shorter blocklength N=512 requires higher $\gamma_{\Delta }$ than longer blocklength N=1024 does. Fortunately, increasing $N_{r}$ reduces this gap and thereby improves spectral efficiency.

### 4.1. mFBT-NOMA with Different FECs

**Figure 8:**
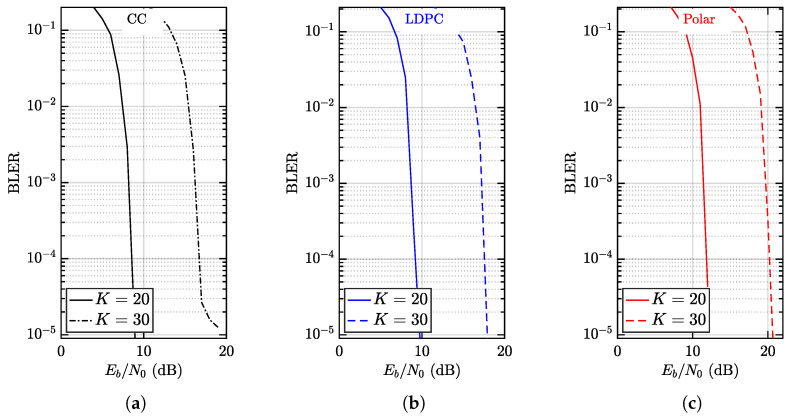
Comparison of GMSK based mFBT-NOMA with different FECs: (**a**) represents CC, (**b**) represents LDPC and (**c**) represents Polar, respectively, where N=512, $N_{r}=6$, and K=20,30. The sum spectral efficiency is thus 10 bps or 15 bps, when K=20 or K=30.

In Figure 8, the BLERs of mFBT-NOMA given different FECs are presented, where $N_{r}=6$, K=20,30 and N=512. It is observed that the target BLER $\epsilon =10^{-5}$ is achievable in all setups, disregarding the FEC employed.

In Figure 8a, the performance of CC based mFBT-NOMA is evaluated in terms of BLER vs. $E_{b}/N_{0}$. Here, each user attains spectral efficiency R=0.5 bps as a result of GMSK modulation, and hence the sum spectral efficiency is 10 bps or 15 bps when K=20 or K=30. Accordingly, the power consumption in terms $E_{b}/N_{0}$ is 9 dB or 19 dB approximately. Though $\mathrm{BLER}=10^{-5}$ is achieved, it is observed that the BLER curve flattens out as approaching $10^{-5}$. From the perspective of coded modulation theory, such behavior is identified as an error floor. The reason is mainly the small free Euclidean distance, which fails to mitigate residual errors; these can be mitigated using more complex CC, as pointed out in [36]. However, ${\left(7,5\right)}_{8}$ is still recommended not only for its comparable or even better performance but also for its low complexity, which is favorable in IoT for reduced power consumption.

In Figure 8b, the performance of LDPC-based mFBT-NOMA is presented. The configuration is similar to CC-based mFBT-NOMA, and hence the achievable sum spectral efficiency is 10 bps or 15 bps when K=20 or K=30. The difference is that LDPC-based mFBT-NOMA requires slightly higher $E_{b}/N_{0}$ than CC-based mFBT-NOMA does as K=20. However, LDPC-based mFBT-NOMA does not manifest an error floor as K=30.

In Figure 8c, the performance of Polar-based mFBT-NOMA is presented. The configuration is similar to CC- and LDPC-based mFBT-NOMAs, and the achievable sum spectral efficiency is again 10 bps or 15 bps when K=20 or K=30. It is observed that Polar-based mFBT-NOMA outperforms CC-based and LDPC-based mFBT-NOMA in terms of $E_{b}/N_{0}$. The apparent gap is approximately 2.5 dB, disregarding *K*, which is a joint result of the lack of CRC and the short blocklength, as noted in [43].

In summary, the results presented confirm the success of the proposed design in supporting massive connectivity in the finite blocklength regime. More interestingly, the success is achieved without resorting to high-complexity FECs, such as LDPC and Polar. This observation recommends CC for mFBT-NOMA-enabled IoT and will be further evaluated in the subsequent discussion, given different setups, where the results reveal that low BLER can be obtained even with increasing connectivity.

### 4.2. Energy Efficiency

**Figure 9:**
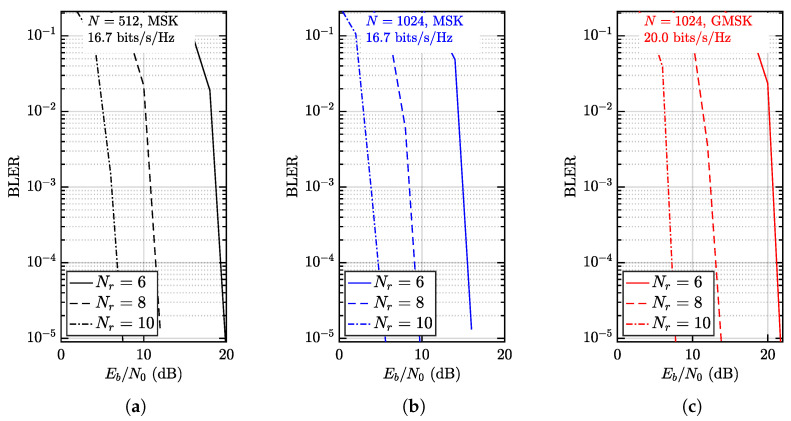
The achievable sum spectral efficiency given CC FEC, as K=40, $N_{r}=6,8,10$, and N=512,1024, where (**a**) represents N=512 using MSK, (**b**) represents N=1024 using MSK and (**c**) represents N=1024 using GMSK. Thus the achievable sum spectral efficiency is 16.7 bps, 16.7 bps and 20.0 bps, respectively.

The diversity gain and energy efficiency given connectivity K=40 are evaluated in Figure 9. As mentioned earlier, the FEC is CC. In Figure 9a, the performance of mFBT-NOMA given N=512 and MSK modulation is demonstrated. The connectivity K=40, such that the system reaches a sum spectral efficiency of 16.7 bps, which is comparable to yet slightly higher than GMSK-based mFBT-NOMA presented in Figure 8a. The BLER performance given $N_{r}=$ 6, 8, and 10 is compared. When $N_{r}=6$, the required $E_{b}/N_{0}$ is approximately 20 dB, which is also similar to GMSK-based mFBT-NOMA, as shown in Figure 8a. When increasing $N_{r}$ to 8 and 10, the improvement is up to 7 dB and 11 dB, respectively. The significant gain constitutes two parts. The first part is increased diversity gain, and the second part is strengthened convergence of $\gamma_{k}$, both of which are cross-verified in Figure 5.

In Figure 9b, the performance of mFBT-NOMA with increased blocklength is evaluated. The configuration is identical to Figure 9a, except for N=1024. It is readily seen that increasing N=512 to N=1024 reduces BLER significantly. For example, the required $E_{b}/N_{0}$ is reduced by ≈4 dB to offer the same sum spectral efficiency while still keeping $\mathrm{BLER}=10^{-5}$ when $N_{r}=6$. Similar behavior appears as well when $N_{r}=8$ and $N_{r}=8$, which is termed interleaving-gain in a coded modulation scheme.

In Figure 9c, the performance of mFBT-NOMA with GMSK modulation is evaluated. The configuration is identical to Figure 9b, except for the GMSK modulation. The diversity and interleaving gains can be readily confirmed. Due to GMSK, the spectral efficiency reaches 20 bps, which is significantly higher than that of MSK-based systems. However, improved spectral efficiency inevitably incurs higher $E_{b}/N_{0}$.

**Figure 10:**
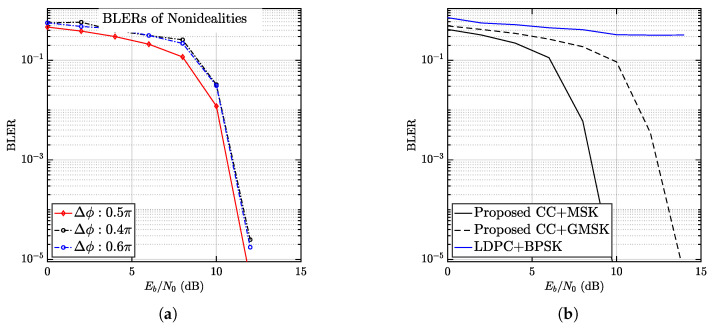
Results concerning (**a**) the nonideal configurations and (**b**) comparison with LDPC+BPSK-based mFBT-NOMA. In both systems, $N_{r}=8$, K=40, and N=1024.

In Figure 10, the performance of mFBT-NOMA is evaluated concerning nonideal configurations and comparison with some linear modulation-based schemes. First in Figure 10a, the impact of Δϕ in (2) is evaluated. Three different cases, i.e., Δϕ=0.5π, 0.4π, 0.6π, where Δϕ=0.5π is optimum according to [42]. The results confirm the optimality of Δϕ=0.5π, seeing an improvement of approximately 0.5 dB over Δϕ=0.4π, 0.6π, while using Δϕ=0.4π or Δπ=0.6π makes very little difference. The explanation can be found in ([42], Figure 3), which shows that the strength of the multiuser interference is identical given Δϕ=0.4π or Δπ=0.6π.

In Figure 10b, the performance of mFBT-NOMA is compared with some representative NOMA schemes employing FECs. In these schemes. BPSK and LDPC are extensively studied and recommended [22,23]. When given $R_{\mathrm{FEC}}=0.5$, LDPC+BPSK offers R=0.5 bps, which is exactly equal to GMSK+CC but higher than MSK+CC, as can be perceived. As the results indicate, the proposed schemes outperform the LDPC+BPSK scheme. There are two main reasons. First, most existing schemes employ an equal-power allocation technique in multi-antenna NOMA, which often fails to guarantee successful detection even with a very low code rate $R_{\mathrm{FEC}}$. The second reason is using Δϕ (2) to aid the mitigation of multiuser interference significantly [42]. By applying the proposed technique in terms of power allocation and Δϕ, as already shown in Figure 8b. Although a detailed comparison is not presented, it is worth noting that the proposed system supports more users than power-domain NOMA, since the maximum number of users is set to $K=2N_{r}$ in principle.

Nevertheless, the proposed mFBT-NOMA offers significantly improved energy efficiency by jointly exploiting diversity and interleaving gains. More importantly, the proposed method, in terms of power allocation and Δϕ, is proven effective in optimizing existing LDPC-based systems.

## 5. Conclusions

The design and performance analysis of multi-antenna-aided NOMA supporting ultra-dense IoT in the finite-blocklength regime are presented. To facilitate URLLC in such a large-scale, overloaded system, a high-dimensional analysis is introduced. Analytical expressions for the attainable SINR and the resulting spectral efficiency in the finite-blocklength regime are derived and subsequently validated numerically. These expressions are then exploited to design URLLC-enabled IoT with special focus on MSK/GMSK signaling, which are employed in a number of IoT standards, including IEEE 802.15.4 and Bluetooth. The simulated results confirm that URLLC and high user load can be simultaneously supported using the proposed design, with CC, LDPC, and Polar, all candidate FECs for 5G and beyond. Interestingly, CC-based systems outperform the other two in that similar performance in terms of $E_{b}/N_{0}$ vs. BLER can all be achieved by three FECs, but employing CC significantly reduces transceiver complexity and, consequently, power consumption. This is an advantageous attribute in IoT for an extended life cycle.

## Figures and Tables

**Table 1:** Parameters of FECs.

FECs	Parameters			
	Block Length N	Decoding	$R_{\mathrm{FEC}}$	Complexity
CC	1024/512	MAP	0.5	Low
LDPC	1024/512	BP	0.5	High
polar	1024/512	SCAN	0.5	Very High

## Data Availability

Restrictions apply to the datasets: The datasets presented in this article are not readily available because the scripts/code are part of an ongoing project which can hardly be open-sourced. Requests to access the datasets should be directed to the corresponding author (wuxinhao@mail.nwpu.edu.cn).
